# Supplementary material for: The interactome of intact mitochondria by cross-linking mass spectrometry provides evidence for coexisting respiratory supercomplexes
Source: Mol Cell Proteomics. 2017 Dec 8;17(2):216–32. doi: 10.1074/mcp.RA117.000470 (PMC5795388; doi:10.1074/mcp.RA117.000470)
Supplement: Supplemental Data [file supp_RA117.000470_133922_0_supp_23978_fzffwf.zip › spectra_annotation/mito_spectra_annotation/196-1-7-1-18-1.pdf]

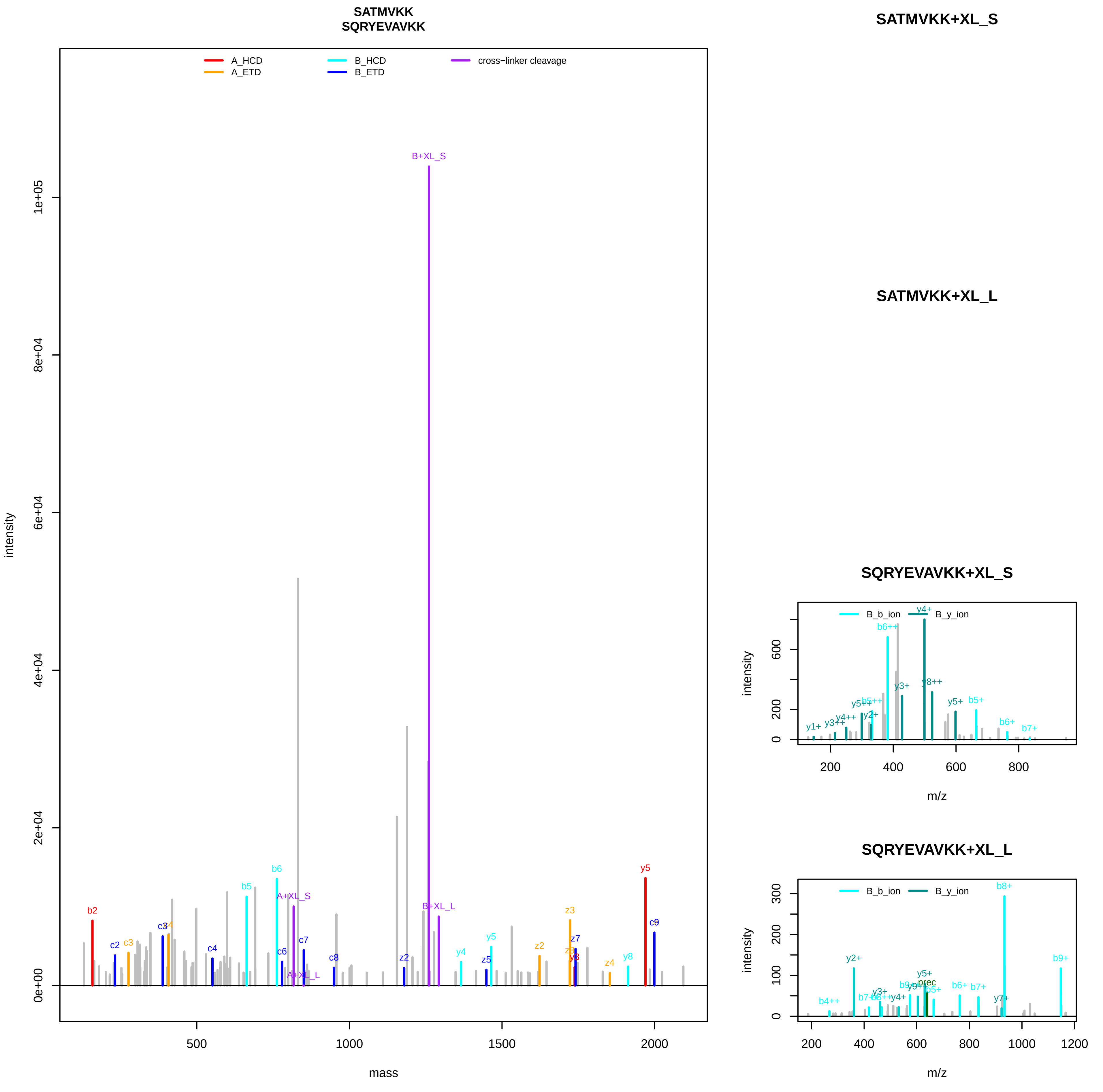

SATMVKK
SQRYEVAVKK
A_HCD
A_ETD
B_HCD
B_ETD
cross−linker cleavage
B+XL_S
A+XL_S
A+XL_L
B+XL_L
b2
c2
c3
c3
c4
b5
b6
c6
c7
c8
z2
y4
z5
y5
z2
z3
z7
y8
z4
y5
c9
intensity
mass
0e+00
2e+04
4e+04
6e+04
8e+04
1e+05
500
1000
1500
2000
SATMVKK+XL_S
SATMVKK+XL_L
SQRYEVAVKK+XL_S
B_b_ion
B_y_ion
y4+
b6++
y8++
y3+
y5++
b5++
y2+
y5+
b5+
b6+
b7+
y1+
y3++
y4++
intensity
m/z
0
200
600
200
400
600
800
SQRYEVAVKK+XL_L
B_b_ion
B_y_ion
b8+
y2+
b9+
y5+
prec
b4++
b7++
b8++
y3+
y4+
b5+
b6+
b7+
y7+
intensity
m/z
0
100
200
300
200
400
600
800
1000
1200
